# Recent advances in understanding transcription termination by RNA polymerase II

**DOI:** 10.12688/f1000research.8455.1

**Published:** 2016-06-23

**Authors:** Travis J. Loya, Daniel Reines

**Affiliations:** 1 Department of Biochemistry, Emory University School of Medicine, Atlanta, GA, 30322, USA

## Abstract

Transcription termination is a fundamental process in which RNA polymerase ceases RNA chain extension and dissociates from the chromatin template, thereby defining the end of the transcription unit. Our understanding of the biological role and functional importance of termination by RNA polymerase II and the range of processes in which it is involved has grown significantly in recent years. A large set of nucleic acid-binding proteins and enzymes have been identified as part of the termination machinery. A greater appreciation for the coupling of termination to RNA processing and metabolism has been recognized. In addition to serving as an essential step at the end of the transcription cycle, termination is involved in the regulation of a broad range of cellular processes. More recently, a role for termination in pervasive transcription, non-coding RNA regulation, genetic stability, chromatin remodeling, the immune response, and disease has come to the fore. Interesting mechanistic questions remain, but the last several years have resulted in significant insights into termination and an increasing recognition of its biological importance.

## Introduction

Transcription termination is the complex and tightly regulated process in which polymerase stops RNA chain elongation and dissociates from the end of transcription units. A multiplicity of termination factors, which assemble into a number of complexes, govern the biogenesis of various types of transcripts including messenger RNA (mRNA), small nuclear RNA, small nucleolar RNA (snoRNA), and long non-coding RNA (lncRNA). Much of what we know has been learned from studies in *Saccharomyces cerevisiae*, where one process operates to terminate short non-coding transcripts and another involves components of the polyadenylation (polyA) machinery and nucleases such as Xrn2 to terminate the synthesis of mRNA precursors. Recent reviews have described some of these fundamental mechanisms and factors involved in termination by RNA polymerase II (pol II) ^1– 6^. Here, we will focus on the role of the termination of transcription by pol II in a variety of biological contexts and describe how new discoveries have helped elucidate longstanding questions as well as contributed to the establishment of new paradigms. It has become clear that transcription termination occupies a critical role as a regulator of cellular processes. This process and the termination machinery occupy an increasingly important place in human health and disease.

## End-of-open reading frame termination: recent studies on the torpedo and allosteric models of termination of mRNAs

Two prevailing hypotheses for how pol II terminates transcription have guided experiments for almost three decades; these are the so-called *allosteric* and *torpedo* models. In its simplest form, the first posits that as elongating pol II encounters a polyA signal, a physical change in the complex is triggered that provokes termination ^7^. The torpedo model suggests more specifically that termination is facilitated by a nuclease brought to the transcript *via* the polyA signal and associated RNA processing machinery. After the co-transcriptional endonucleolytic cutting of the primary transcript in preparation for its polyadenylation, a nuclease engages the 5’ end of the 3’ portion of the RNA that remains polymerase associated, and digestion of this RNA ‘stump’ in the 5’ to 3’ direction enables the nuclease to chase down pol II, whereupon termination is triggered by an undetermined mechanism ^8^. The difference between these two models may be reduced to the following question: what are the changes in pol II that persuade it to go from its default mode of continuing elongation into stopping and departing from the template? Although there has been some controversy over the relative acceptance of these two hypotheses, the two models are not mutually exclusive, and indeed a combination of both mechanisms has been proposed ^9– 11^.

A recent test of the concept provides new insight showing that *in vitro*, a polyA signal is sufficient to induce elongation complex disassembly independent of transcript cleavage ^12^. These authors propose that there is an important conformational shift that can be blocked by α-amanitin, implicating pol II itself in this transition. These findings seemingly support an allosteric component to the model, *i.e.* something happens to pol II following synthesis of the polyA signal in the nascent RNA, but cutting of the RNA is not needed. However, identifying that change in pol II has remained elusive. In an almost reciprocal study, it had been shown that exonucleolytic degradation of the transcript by the Rat1/Rai1 nuclease (the two subunits that compose yeast Xrn2) was found to be insufficient to dislodge pol II from its template *in vitro* ^10, 13^. So, even if a nuclease torpedo is involved in termination, it is not sufficient to complete the process.

The conserved Xrn2 nuclease has been considered a strong candidate to be the torpedo. Recently, Fong *et al*. showed that Xrn2 was necessary for proper termination at thousands of genes ^14^. The requirement was not absolute in that loss of Xrn2 delayed, rather than prevented, termination, resulting in longer transcripts. This supports the idea of a kinetic competition in termination, as the Xrn2 torpedo chases the elongating polymerase while Xrn2 digests the transcript from 5’ to 3’. In cells with mutant pol II that was either abnormally slow or fast, it should take Xrn2 less and more time to catch pol II, respectively. Consistent with this prediction, slow pol II led to shorter terminated transcripts, and fast pol II produced longer terminated transcripts. This result can be taken as strong evidence in favor of the torpedo model. Curiously, Xrn2 was found to be important for termination, even for non-coding transcription units whose miRNA and lncRNA products are not acted upon by the cleavage and polyA machinery.

In another test of the need for cleavage of the primary transcript for termination, Schaughency *et al*. mapped the chromatin locations of pol II in a *S. cerevisiae* strain depleted for Ysh1, the endonuclease that generates the 3’ end that becomes polyadenylated ^11^. The analysis revealed that pol II stops elongation but remains template bound, presumably because the lack of cleavage prevents a nuclease torpedo from gaining access to the elongation complex ^11^. This gives rise to the idea that both an allosteric change resulting from crossing the polyA site and the torpedo effects of Rat1 are necessary for efficient pol II release in yeast.

While implicated as an essential part of the torpedo model, there is also a growing body of research on Xrn2’s post-translational modification. Sansó *et al*. ^15^ employed a chemical genetic screen introduced by Shokat and co-workers ^16, 17^ to look for substrates of the Cdk9 kinase, an enzyme implicated in transcription as a subunit of the pTEFb elongation factor ^18^. By mutating its active site to accommodate a bulky ATP analog, these investigators could label kinase substrates specific for Cdk9 that could then be tracked by their covalently attached thiophosphate group. One such substrate was Xrn2 ^15^. The phosphorylated form of Xrn2 could be found on chromatin and the modification was associated with a modest enhancement of its nuclease activity. Inhibition of Cdk9 kinase activity and mutation of the phosphorylated threonine in Xrn2 led to the expected defects in termination predicted by the torpedo model. This new finding incorporates Xrn2 into a cellular signaling pathway. It will be important to learn what cellular events activate and reverse phosphorylation-based control of the torpedo’s activity.

Xrn2 was also recently shown to regulate chromatin structure to promote meiotic gene silencing during vegetative growth in *Schizosaccharomyces pombe* ^19, 20^. In this case, heterochromatin formation was dependent on transcription and termination, which was coupled to the nucleolytic elimination of the resulting RNAs. Chalamcharla *et al*. showed that the Xrn2 homolog Dhp1 was necessary for premature termination of non-coding RNAs that marked sites of repression of facultative heterochromatin at meiotic genes ^19^. Loss of *dhp1* in an exosome-deficient strain resulted in compromised RNAi-mediated gene silencing, similar to the changes found in *ago1* mutants. Tucker *et al.* also found a similar effect for Dhp1 in silencing and also showed that loss of Dhp1 resulted in defects in meiotic chromosome segregation ^20^. Interestingly, the two groups have different mechanistic explanations for their observations. Chalamcharla *et al.* postulate that termination-coupled degradation by the exosome triggers recruitment of the heterochromatin machinery. Tucker *et al.* consider the functions of the Dhp1 nuclease in termination and gene silencing as separable, with the termination role not an important aspect of its silencing duty. These reports, and that of Kowalik *et al*. ^21^, have been productive investigations into the role of transcription termination factors in *S. pombe* gene silencing, a phenomenon that had been explored previously in *S. cerevisiae* ^22– 24^ but which seems mechanistically distinct between the yeast species considering the absence of post-transcriptional gene silencing in *S. cerevisiae*.

## Pervasive transcription and termination

In recent years, it has been repeatedly shown that transcription initiation is promiscuous and widespread, with much of the genome capable of being copied into RNA. A corollary to this is the recognition that the termination reaction is an important governor over the transcriptome in its capacity to partition or funnel transcription products into a degradative ‘clean up’ pathway for RNA elimination (such as seen for cryptic unstable transcripts), a maturation pathway for limited processing (such as snoRNAs), or an option to yield spliced and polyadenylated mRNAs.

One form of pervasive transcription is seen as divergent initiation from promoters. Recent work has gone into characterizing transcripts that radiate in both directions from bidirectionally firing promoters ^25– 29^. Non-coding transcripts that extend in the ‘wrong’ (antisense) direction are terminated, and their degradation is tightly coupled to this process ^30– 34^. Interestingly, the genomic DNA extending in that direction tends to be enriched in polyA signals and hence termination sequences. In contrast, those in the sense or mRNA-yielding direction are depleted of termination potential because they are enriched in splicing signals, which protect transcription from premature termination ^35, 36^. Thus, termination enforces promoter directionality.

A recent genome-wide study showed that prematurely terminated transcripts are one class of RNA that is cleaned up by nuclease surveillance. Mutation of the human nuclear ribonuclease complex, known as the exosome, revealed the surprising frequency with which prematurely terminated RNAs are generated from the genome ^37^, something that might be expected given the imperfect processivity of pol II engaged in transcribing megabase-long genes *.*

In an interesting flipping of the conventional way of thinking that termination feeds transcripts to the degradation machinery, a study in *S. pombe* suggests that the exosome complex can capture a specific paused and backtracked form of pol II and take it to the termination pathway ^38^. Here, the exosome is thought to directly recognize the 3’ end of the extruded nascent transcript in arrested (‘backtracked’) complexes. Yet unresolved is whether the protein sets employed in conventional (polyA-coupled) pol II termination or short non-coding termination (such as the Nrd1-Nab3-Sen1 system in *S. cerevisiae*) are required for this mode of stopping transcription.

Not only is the regulation of termination important for cellular genomes but also viruses can antagonize normal transcription termination during infection. Using 4-thiouridine labeling of RNA and ribosome profiling of newly made RNA following infection, Rutkowski *et al*. found that herpes virus inhibits the termination of host, but not viral, transcription ^39^. The resulting readthrough RNAs became observable in a manner similar to those seen in other systems when the exosome or termination machineries become incapacitated by experimental manipulation ^24, 28, 37^. These aberrant, even polygenic, transcripts were not spliced or translated properly, thus resulting in a form of host shut-off that may favor viral gene expression. How widespread the phenomenon of viral corruption of the integrity of the host’s transcription units is, and its mechanistic basis, remains to be elucidated.

Another case of a pathologically defeated transcription termination system was observed by Grosso *et al*. in a human cancer ^40^. Through transcriptome profiling of various renal cell carcinoma samples, they found widespread transcriptional readthrough in many of the samples from patients with some of the worst prognoses. Again, chimeric transcripts spanning transcription units could be identified. The *SETD2* gene, which encodes a histone methyltransferase, was frequently inactivated in these cancer lines. Knockdown of *SETD2* could recreate the readthrough phenotype. Ectopic expression of the wild-type protein could reverse it, thus implicating chromatin modifications in disease-related failure to terminate effectively. Interestingly, Bcl2, an anti-apoptotic protein often elevated in cancer, was upregulated, suggesting a possible mechanism by which mutation leads to loss of proliferative control.

Yet another version of a hybrid readthrough transcript called DoGs ( downstream of genes containing transcripts) has been identified ^41, 42^. These transcripts have a 5’ end corresponding to an upstream gene and an aberrant 3’ extension of up to tens of kilobases. Hundreds of DoGs were detected following KCl treatment of neuroblastoma cells whereupon their length and abundance increased. These readthrough products, only some of which became polyadenylated, encountered fewer polyA sites downstream of the coding sequence, which could explain less efficient termination for the DoG transcript. It is not clear what functional role the DoGs play; their strong association with chromatin suggests they become incorporated into the nuclear scaffolding during stress responses. Intuitively, this is an appealing model, as osmotic stress shrinks the cell nucleus and collapses chromatin. DoGs may mitigate such deleterious effects, as recently suggested for a set of non-coding, repeat-containing RNAs ^43^. This finding emphasizes that terminator override plays an important role across physiological states and reveals plasticity in our definition of a transcription unit.

The continued accumulation of instances of pervasive transcription has changed our view of cellular RNA from one that is ‘open reading frame centric’ to one with a greater appreciation for RNA arising from intergenic sequences, either constitutively or under specific physiological or pathological conditions.

## Transcription termination in immunological systems

The transcription elongation complex has been of interest to immunologists for many years owing to transcription-dependent mutations introduced into immunoglobulin (Ig)-encoding genes during somatic hypermutation ^44^. Somatic hypermutation is the process by which the variable regions of Ig genes change as the immune response matures, leading to selected antibodies with increasingly higher affinities. A mutagenic enzyme, activation-induced cytidine deaminase (AID), is thought to track with elongating pol II and is associated with premature termination of transcription. This mechanism is proposed to link transcription to a specific hotspot for physiologically important mutations. A recent report extends this model and proposes that pervasive transcripts are functionally important for proper class switch recombination and somatic hypermutation in B lymphocytes ^45, 46^. The suggested model is that transcripts in paused, divergently facing elongation complexes become stabilized, offering the complex more of an opportunity to form short DNA-RNA hybrids called R-loops. These loops are part of the transcription termination reaction in which RNA is hybridized to the DNA from which pol II has just departed and can be considered an intermediate on the pathway to disintegration of the transcription bubble and the digestion of the short, anti-sense RNA. B lymphocytes appear to have evolved a mechanism to capture this nucleic acid framework with its exposed single-stranded DNA and make it a target for the AID mutagenesis machinery. In this manner, B cells can focus the genetic changes on the very specific region of Ig proteins that need to vary. We will come back to a discussion of the importance of R-loops in termination in the next section.

Wang *et al*. also present evidence in support of the idea that premature termination provides an opportunity for the single-stranded DNA of the transcription bubble to become a substrate for localized mutation by AID ^47^. They show *via* chromatin immunoprecipitation that pol II concentrates in the region that undergoes hypermutation. Subsequent manipulation of elongation factor Spt5 by knockdown, which should facilitate premature termination, revealed that DNA strands in the region acquire a single-stranded character and are mutated at higher frequency. Early termination was suggested by the molar abundance of RNA from the 5’ variable region of the heavy chain locus *versus* the downstream sequences.

In another immunologically interesting system, the Zfp318 protein has been postulated to regulate termination site selection during the transcription of the gene that encodes both the μ and δ heavy chains in B lymphocytes ^48^. Typically, a precursor transcript is synthesized that encodes the μ heavy chain constant region exons and, further downstream, the corresponding δ constant region exons. Alternative splicing appends one or the other sets of exons to the VDJ-encoding exons, thereby yielding mRNAs for complete IgM or IgD heavy chains. This regulatory event has been known for some time, although the responsible *trans*-acting factors have been elusive ^49, 50^. By engineering a conditional Zfp318 deficiency into the bone marrow lineage of mice, Pioli *et al*. showed that Zfp318 normally serves to repress an apparent transcriptional termination event at the end of the μ exon series. Thus, there is normally some readthrough into the δ exons, thereby generating a primary transcript that can yield either μ- or δ-encoding mRNA, depending on the splicing pattern ^48^. In the absence of Zfp318, it is suggested that the termination event becomes so strong that transcripts hardly extend into the δ exons, leaving cells with a pool of precursor transcripts ending near the polyA site for the upstream μ exons, and giving B cells no option of making IgD. This was one of the few shifts observed in that transcriptome, showing a very specific consequence following the loss of Zfp318. An independent set of experiments confirmed that incapacitating Zfp318 resulted in a shift from IgD and IgM production to mainly IgM production ^51^. Enders *et al*. attributed this effect to the rate of pol II elongation and a change in the efficacy of competition between polyadenylation at the end of the μ exons *vs*. alternative splicing around them to append the δ exons. It will be interesting to learn how the Zfp318 protein, which may bind nucleic acids and could be a key regulator of this process, operates and if it directly influences a true transcription termination event.

## R-loops, dicer, and genomic stability

The role of the R-loop in somatic hypermutation and recombination associated with heavy chain class switching is a specialized use of a common feature of the transcription termination zone. One hypothesized function of the senataxin protein, which is the human orthologue of the Sen1 termination factor initially discovered in yeast, is to provoke termination by unraveling the R-loop using a helicase activity ^1, 52^. However, these structures may also be involved in coordinating chromatin remodeling, as elucidated in a recent study in which repressive chromatin marks were induced over terminators ^53^. In one example, an RNA duplex is suggested to form from anti-sense transcription of the R-loop, which recruits dicer to the region, leading to trimethylation of H3K9 at the termination zone, thereby linking the termination system to chromatin and the RNAi system in human cells.

Interestingly, Castel *et al.* show a role for Dicer1 in termination for all three nuclear polymerases, but this function appears to be independent of the rest of the RNAi machinery in *S. pombe* ^54^. *dcr1Δ* cells show increased polymerase occupancy at the end of genes, implying stalling of polymerase. Deletion of other RNAi machinery or generating a catalytically dead Dicer1 mutant did not give similar results, suggesting the process is not RNAi mediated. The genes regulated by this mechanism seem to be restricted to those at sites of replicative stress, where high transcription levels result in collision of the transcription bubble and the replication fork. Since DNA-RNA hybrids at these sites are recombinogenic, Dcr1 may be important for maintaining genome integrity, similar to the way senataxin resolves the problem in mammalian cells, although the latter is thought to do so through a helicase activity ^55, 56^. The role for dicer in termination could also be related to prior findings that Rnt1, another RNAse III-type enzyme in *S. cerevisiae*, can provoke termination ^57^.

R-loop-mediated termination is also proposed to play a role in Friedrich’s ataxia. It was suggested that R-loops aberrantly form in the frataxin gene as a result of expansion of GAA repeats in its first intron ^58, 59^. Mutated frataxin exhibits features of heterochromatin, H3K9 methylation, and decreased acetylation of H3 and H4, thus a reduced level of expression is to be expected. However, the altered gene also shares many characteristics of canonical R-loop-terminated genes, including a polyA-signal-like sequence upstream of the expansion, followed by a GU-rich sequence similar to the downstream element of polyA signals. This has led to a proposal that the mutated frataxin allele is the victim of premature termination, which contributes to its low level of expression in patients ^59^. While experimental verification is still needed, this is an interesting model for a role of termination in disease.

In a final case of R-loop involvement in termination, Zhao *et al*. studied the modification of the repeat region of pol II’s largest subunit ^60^. They found that dimethylation of a specific arginine serves to recruit the survival of motor neuron (SMN) protein to the elongation complex. This protein, in turn, associates with senataxin, which resolves R-loops and recruits the torpedo nuclease Xrn2 during termination. Both SMN and senataxin have been found to suffer mutations in neurodegenerative diseases, again highlighting the importance of the termination reaction in human health and the imperative to understand its molecular basis.

Our understanding of the role of transcription termination across biological systems has expanded significantly with recent advances in experimental techniques and with growing interest in the topic across biological systems. New findings have yielded fresh insight into the longstanding questions of termination mechanism and somatic hypermutation and have provided new paradigms with the identification of cases in which the stringency of termination is relaxed during specific regulatory events or accidentally in pathological examples of dysfunction.

## Abbreviations

AID, activation-induced cytidine deaminase; DoGs, downstream of genes containing transcripts; Ig, immunoglobulin; lncRNA, long non-coding RNA; mRNA, messenger RNA; pol II, RNA polymerase II; polyA, polyadenylation; SMN, survival of motor neuron protein; snoRNA, small nucleolar RNA.
